# Extra-Renal Elimination of Uric Acid via Intestinal Efflux Transporter BCRP/ABCG2

**DOI:** 10.1371/journal.pone.0030456

**Published:** 2012-02-10

**Authors:** Atsushi Hosomi, Takeo Nakanishi, Takuya Fujita, Ikumi Tamai

**Affiliations:** 1 Department of Membrane Transport and Biopharmaceutics, Faculty of Pharmacy, Institute of Medical, Pharmaceutical, and Health Sciences, Kanazawa University, Kanazawa, Japan; 2 College of Pharmaceutical Sciences, Ritsumeikan University, Kusatsu, Japan; National Cancer Institute, United States of America

## Abstract

Urinary excretion accounts for two-thirds of total elimination of uric acid and the remainder is excreted in feces. However, the mechanism of extra-renal elimination is poorly understood. In the present study, we aimed to clarify the mechanism and the extent of elimination of uric acid through liver and intestine using oxonate-treated rats and Caco-2 cells as a model of human intestinal epithelium. In oxonate-treated rats, significant amounts of externally administered and endogenous uric acid were recovered in the intestinal lumen, while biliary excretion was minimal. Accordingly, direct intestinal secretion was thought to be a substantial contributor to extra-renal elimination of uric acid. Since human efflux transporter BCRP/ABCG2 accepts uric acid as a substrate and genetic polymorphism causing a decrease of BCRP activity is known to be associated with hyperuricemia and gout, the contribution of rBcrp to intestinal secretion was examined. rBcrp was confirmed to transport uric acid in a membrane vesicle study, and intestinal regional differences of expression of rBcrp mRNA were well correlated with uric acid secretory activity into the intestinal lumen. Bcrp1 knockout mice exhibited significantly decreased intestinal secretion and an increased plasma concentration of uric acid. Furthermore, a Bcrp inhibitor, elacridar, caused a decrease of intestinal secretion of uric acid. In Caco-2 cells, uric acid showed a polarized flux from the basolateral to apical side, and this flux was almost abolished in the presence of elacridar. These results demonstrate that BCRP contributes at least in part to the intestinal excretion of uric acid as extra-renal elimination pathway in humans and rats.

## Introduction

Uric acid is a final product of purine nucleoside metabolism in humans, and it is thought that its level is well controlled, mainly by the balance between production in liver from purine nucleosides and excretion into urine. Although its physiological role is poorly understood, uric acid is thought not only to protect neuronal cells due to its antioxidant activity, but also to play a role in maintaining blood pressure [1], [2]. It has been suggested that serum uric acid (SUA) should be kept below 7 mg/dL to prevent hyperuricemia, which is a clinically important risk factor for cardiovascular diseases, chronic kidney disease and gout [3], [4]. In addition, it is known that several drugs in clinical use alter the level of SUA. For example, angiotensin II receptor blockers such as losartan decrease SUA level, while others increase it [5]–[8]. Salicylic acid causes an increase in SUA level at low dose, but a decrease at high dose [9]. Accordingly, it is important to clarify the mechanisms that control SUA level in order to understand the effects of drugs on uric acid disposition and to find better ways of regulating SUA.

Uric acid handling in the kidney is very complex, involving glomerular filtration, tubular reabsorption and secretion. The uric acid transporters URAT1 and URATv1, located at the apical and basolateral membranes of the proximal tubular cells, respectively, are involved in renal reabsorption of uric acid [8]–[13]. On the other hand, organic anion transporters including OAT1 [14], OAT2 [15], [16], and OAT3 [17] at the basolateral membrane and other transporters such as BCRP [18], NPT1 [19], [20], NPT4 [21] and MRP4 [22] at the apical membranes have been reported to be involved in renal secretory transport of uric acid. Among them, BCRP, NPT1 and NPT4 may make significant contributions to renal handling of uric acid, because genetic polymorphisms of these transporters are associated with gout and/or hyperuricemia [13], [23].

Uric acid is also excreted from the body extra-renally, although urinary excretion is predominant. Nevertheless, it has been suggested that one-third to one-fourth of uric acid is recovered in feces, indicating that biliary and/or intestinal secretion is an important alternative pathway(s) of uric acid excretion [24], [25]. However, there has been little mechanistic study on extra-renal excretion of uric acid. Accordingly, the purpose of the present study is to examine the mechanism(s) of uric acid excretion via liver and intestine, focusing on hepatobiliary excretion and intestinal secretion directly from blood into the lumen. Although there are significant species difference in uric acid handling between human and animals, transporters such as URAT1 [10], [26], [27], URATv1 [9], [28], BCRP [29], [30], OATs [14]–[17], [31] and NPTs [19]–[21], [32] are expressed in both humans and rats. However, rats exhibit a significantly lower SUA level than humans owing to the contribution of uricase, which metabolizes uric acid to allantoin as a final metabolic product of purine; this is different from the situation in humans [33]. However, the uricase inhibitor oxonate can decrease uricase activity *in vivo* [34]. Indeed, the SUA level of oxonate-treated rats is increased to a level similar to that seen in normal humans. Accordingly, in the present study, we used oxonate-treated rats to estimate the *in vivo* contribution of extra-renal excretion of uric acid. Furthermore, Caco-2 cells, a well-established model of human intestinal epithelial cells, were used as a model to examine the human intestinal transport mechanism of uric acid.

BCRP/ABCG2 (breast cancer resistance protein) is abundantly expressed at the apical membrane of small intestinal epithelial cells and in liver, and impaired BCRP function is associated with an increase of SUA level [13]. Therefore, in the present study, we focused on the contribution of BCRP to extra-renal clearance of uric acid.

## Results

### *In vivo* excretion of externally administered [^14^C]uric acid in oxonate-treated rats

To estimate the relative contributions of excretory organs, we measured the amounts of recovered radioactivity in urine, bile, and intestinal luminal contents after intravenous administration of [^14^C]uric acid to oxonate-treated rats with bile duct ligation. The results (percentage of dose) are shown in Table 1. Recoveries of radioactivity in urine, bile, and intestinal luminal contents were 42.6, 0.68 and 8.90% of the dose, respectively. Accordingly, urinary excretion was predominant and intestinal secretion was also significant, while biliary excretion was very low. Although the recovery of radioactivity was not complete, this could be explained by retention of uric acid in the animal body and/or elimination via other routes, such as in expelled air following metabolism in the gut lumen [24]. A rough estimation based on the distribution volume obtained from the plasma concentration-time curve and plasma concentration at the time of measurement (2 hrs after administration) suggested that 4.55% of the dose remained in the body.

**Table 1:** Recovery of [^14^C] uric acid up to 120 min in oxonate-treated rats.

C_p_×V_d_	Urine	Bile	Intestine	Bile+Intestine
4.55%	42.58%	0.68%	8.90%	9.58%

Recovery of [^14^C]uric acid was determined in oxonate-treated rats after intravenous administration of [^14^C]uric acid (2 µCi/rat). Each value is the mean of 4 to 9 measurements.

### Secretion of endogenous uric acid into intestinal lumen in oxonate-treated rats

**Figure 1:**
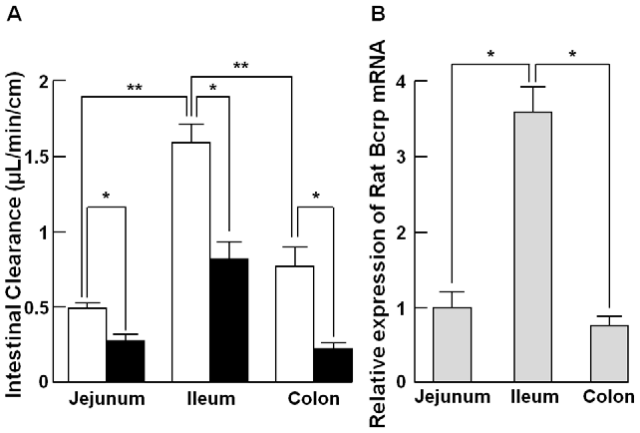
Intestinal secretion of uric acid and regional difference of Bcrp-mRNA expression. (A) Intestinal clearance of uric acid at each of jejunum, ileum, and colon was determined by means of the *in situ* intestinal closed loop method in oxonate-treated rats in the absence (Control, open bars) and presence of elacridar (10 µM, closed bars). Each bar indicates the mean ± S.E.M. (n = 3–8). An asterisk (*) shows a significant difference from the control by Student's t-test (*p*<0.05), and double asterisks (**) show a significant difference from ileum by Student's t-test (*p*<0.05). (B) Level of BCRP mRNA expression was determined in different region of intestine in oxonate-treated rats, and then normalized to rat Gapdh. Each bar indicates the mean ± S.E.M. (n = 6). Expression intensity in jejunum was individually set to 1 and the intensities of ileum and colon were calculated relative to it. An asterisk (*) shows a significant difference from ileum by Student's t-test (*p*<0.05).

Direct intestinal luminal secretion of endogenous uric acid was evaluated in closed loops prepared separately from jejunum, ileum, and colon of oxonate-treated rat (Fig. 1A). Uric acid was directly secreted into the intestinal lumen at all segments, and the values of intestinal secretion clearance were 0.49±0.03, 1.58±0.13, and 0.76±0.17 µL/min/cm loop, respectively. Thus, secretory clearance was in the order of ileum>colon>jejunum, showing a clear regional difference.

### Clearance of endogenous uric acid in oxonate-treated rats

To compare the significance of direct secretion of uric acid from blood to intestinal lumen with excretion into urine and bile, endogenous uric acid clearance was determined in oxonate-treated rats. As shown in Table 2A, urinary and biliary secretory clearances were 0.50 and 0.02 mL/min, respectively, and estimated total intestinal secretory clearance was 0.15 mL/min based on the result of intestinal closed loop assay and intestinal length (Table 2B), assuming that sections of the intestine that were not measured exhibited equal clearance ability. Accordingly, endogenous uric acid is also secreted in a significant amount via the intestine, in the same way as externally administered [^14^C]uric acid (Table 1).

**Table 2:** Clearance of endogenous uric acid in oxonate-treated rats.

CL_renal_	CL_bile_	CL_intestine, estimated_
0.50±0.06	0.02±0.002	0.15

Clearance of endogenous uric acid was measured in oxonate-treated rats. Urine, bile and blood were collected up to 60 min and clearances were calculated by dividing the amount excreted into each part by plasma AUC. Estimated intestinal clearance was obtained as described in Materials and methods using mean value of 5 individual measurements; 118±2.52 cm for length for jejunum and 15.7±0.33 cm for colon, respectively. Values indicate mean ± S.E.M. (n = 5).

### Identification of transporters involved in intestinal secretion of uric acid

**Figure 2:**
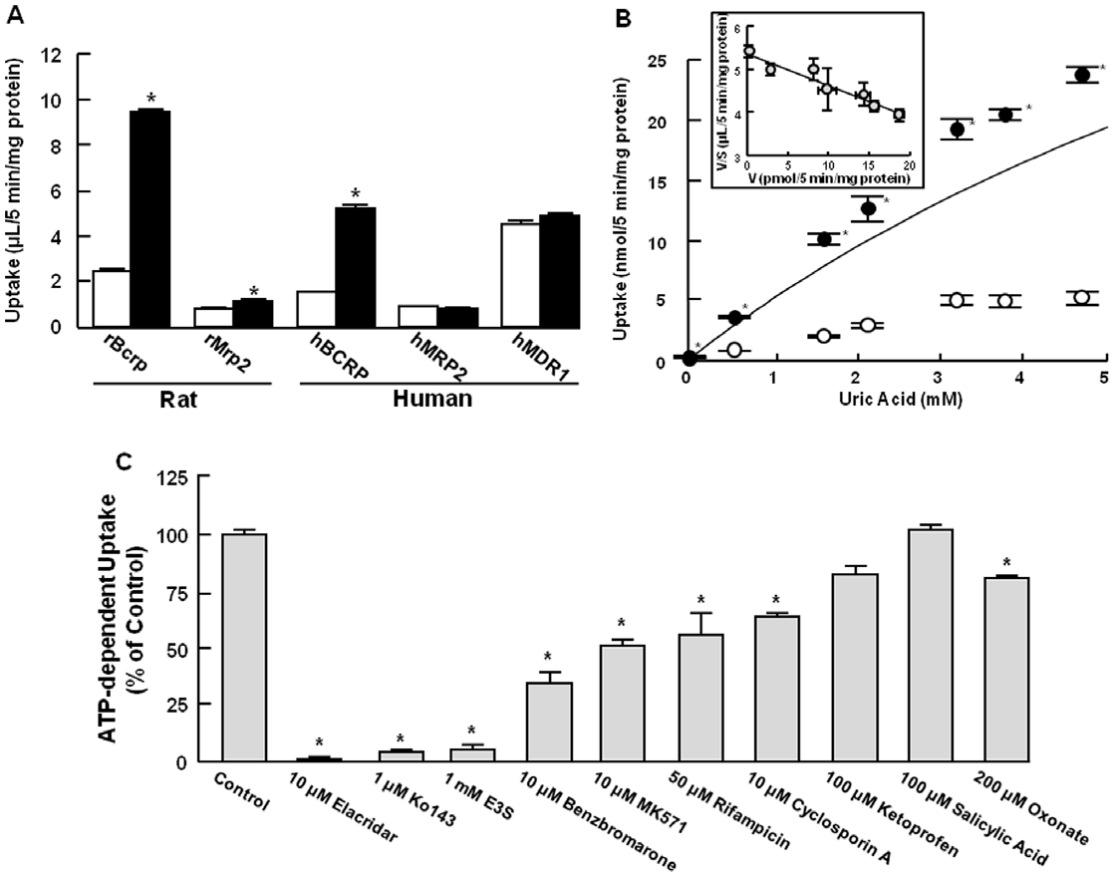
Uric acid transport by ABC transporters. (A) Uptake of [^14^C]uric acid (20 µM) by vesicles expressing rat and human ABC transporters was measured for 5 min at 37°C in the presence of 4 mM ATP (closed symbols) or AMP (open symbols). (B) Concentration dependency of and (C) inhibition study for rBcrp-mediated uptake were studied. Inhibition study was performed in the absence or the presence of various inhibitors at indicated concentrations. ATP-dependent uptake was obtained by subtracting uric acid uptake in the presence of AMP from that in the presence of ATP, and the solid line was drawn by fitting based on non-linear least-squares regression analysis. Saturable transport of uric acid is also shown as an Eadie-Hofstee plot. Each bar shows the mean ± S.E.M. (n = 3–6). An asterisk (*) shows a significant difference from (A and B) uric acid uptake in the presence of AMP and (C) uric acid uptake in the absence of inhibitors by Student's t-test (*p*<0.05).

In order to identify the transporter molecules responsible for intestinal secretion, transport of uric acid was directly evaluated using membrane vesicles expressing human (h) and rat (r) transporters hBCRP/rBcrp, hMRP2/rMrp2, and hMDR1. As shown in Fig. 2A, in hBCRP- and rBcrp-expressing membrane vesicles, uptake of uric acid in the presence of ATP was significantly higher than that in the presence of AMP. Slight ATP-dependent uric acid uptake was observed with rMrp2, whereas hMRP2 and hMDR1 were inactive (Fig. 2A). These results indicated that uric acid is a substrate of rBcrp as well as hBCRP, and also, to a minor extent, rMrp2. Subsequently, uric acid transport by rBcrp was further characterized. Saturation kinetics determined K_m_ value and V_max_ as 12.5±0.77 mM and 67.3±4.21 nmol/5 min/mg protein, respectively (Fig. 2B), although maximum concentration of uric acid was 5 mM due to its low solubility. Low affinity of uric acid to rBcrp which was characterized K_m_ value of 12.5 mM, was very comparable to that to hBCRP (K_m_ = 8.24 mM). Additionally, rBcrp showed similar sensitivity to inhibitors with human BCRP as shown in Fig. 2C.

**Figure 3:**
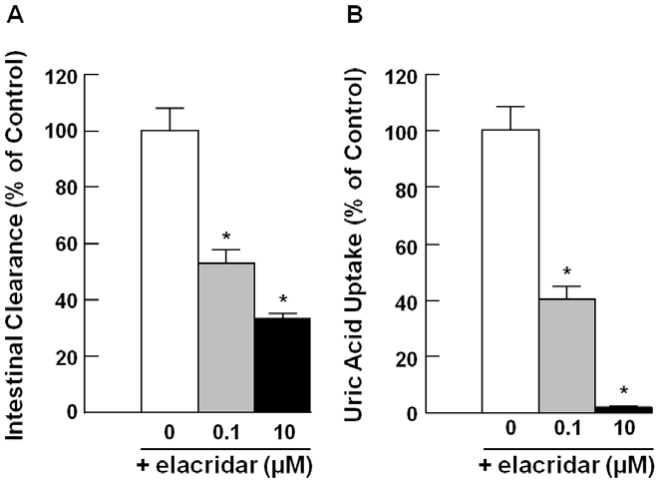
Effect of elacridar on intestinal secretion and rBcrp-mediated uric acid uptake. (A) Intestinal clearance of uric acid at the ileum was determined by means of the *in situ* intestinal closed loop method in the absence (Control, white bar) and presence of 0.1 (gray bar) and 10 µM elacridar (black bar). Each bar indicates the mean ± S.E.M. (n = 4–8). An asterisk (*) shows a significant difference from the control by Student's t-test (*p*<0.05). (B) Uptake of [^14^C]uric acid (20 µM) by membrane vesicles was measured for 5 min at 37°C in the absence (Control, open bar) and presence of 0.1 (gray bar) or 10 µM elacridar (closed bar). Each bar shows the mean ± S.E.M. (n = 3–6). An asterisk (*) shows a significant difference from uric acid uptake in the absence of elacridar by Student's t-test (*p*<0.05).

To further determine if rBcrp contributes to intestinal secretion of uric acid in rats, the influence of an inhibitor of hBCRP/rBcrp, elaclidar [35], on uric acid uptake by rBcrp-expressing membrane vesicles and intestinal secretion was examined by the *in situ* closed ileal loop method. Because MDR1 does not transport uric acid, altered transport of uric acid by elacridar, which is a strong inhibitor for both MDR1 and BCRP, can be attributed to its inhibitory effect on BCRP activity (Fig. 2A). The results are shown in Figs. 1A and 3. In the presence of 10 µM elacridar, intestinal secretory clearance was significantly decreased to 0.27±0.04 (55% of that in elacridar-untreated rats), 0.82±0.12 (52%), and 0.23±0.04 (30%) µL/min/cm loop in jejunum, ileum, and colon, respectively (Table 1A). Interestingly, the regional difference of intestinal secretion of uric acid tended to be associated with the regional difference of mRNA expression levels of rBcrp, which was most highly expressed in ileum (Fig. 1B). Furthermore, ileal secretion of uric acid was inhibited to 53% and 34% of the control in the presence of 0.1 and 10 µM elacridar, respectively (Fig. 3A). Such an inhibitory effect of elacridar on ileal secretion of uric acid was comparable to the effect of elacridar on uptake of uric acid by membrane vesicles expressing rBcrp (Fig. 3B). Accordingly, rBcrp is likely involved in uric acid secretion into the intestinal lumen.

### Plasma uric acid and intestinal clearance in oxonate-treated wild-type and Bcrp1 knockout mice

**Figure 4:**
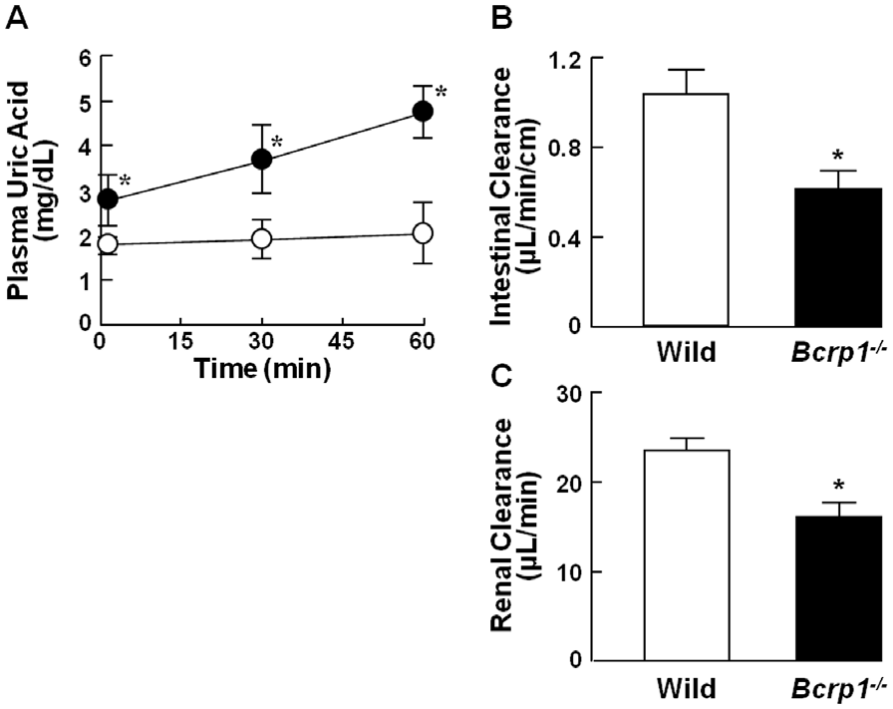
Plasma uric acid concentration and its intestinal and renal clearance in oxonate-treated mice. (A) Plasma uric acid concentration, (B) intestinal clearance at the ileum, and (C) renal clearance were measured in oxonate-treated wild-type (open symbols) and *Bcrp1^−/−^* (closed symbols) mice by means of the intestinal closed loop method and metabolic cages. Each value indicates the mean ± S.E.M. (n = 4–5). An asterisk (*) shows a significant difference from wild-type mice by Student's t-test (*p*<0.05).

To examine the *in vivo* contribution of Bcrp1 to intestinal secretion of uric acid, we measured endogenous uric acid in plasma and evaluated its intestinal secretion by means of the intestinal closed loop method in wild-type and Bcrp1-knockout (*Bcrp1* ^−/−^) mice. As shown in Fig. 4A, plasma uric acid level was much higher in *Bcrp1* ^−/−^ mice compared with wild-type mice after oxonate treatment. In addition, the intestinal secretory (0.61±0.08 µL/min/cm) and renal clearance (16.0±1.77 µL/min) of endogenous uric acid in *Bcrp1* ^−/−^ mice was significantly lower than those in wild-type mice (1.04±0.11 µL/min/cm, and 23.5±1.42 µL/min), respectively (Fig. 4B, C). Accordingly, it was demonstrated that Bcrp contributes at least in part to the intestinal secretion of uric acid.

### Transcellular transport of uric acid in Caco-2 cells

To examine whether uric acid is secreted into gut lumen via BCRP in humans, we studied transcellular transport of uric acid in Caco-2 cells (Table 3). Secretory transport of uric acid across Caco-2 cell monolayer was more than two-times greater than absorptive transport (1.73±0.11 vs. 0.81±0.02 (×10^−6^ cm/sec)), and such polarized transport was significantly reduced in the presence of elacridar (an inhibitor for both MDR1 and BCRP), Ko143 (a potent inhibitor for BCRP) and MK571 (an inhibitor for MRPs), but not verapamil (an inhibitor for MDR1). Accordingly, secretory transport is thought to be predominantly due to BCRP in humans because uric acid is unlikely a substrate of MDR1 and MRP2 (Fig. 2A).

**Table 3:** Transcellular transport and the effect of elacridar on uric acid transport in Caco-2 cells.

Condition	Permeability (×10^−6^ cm/sec)		Efflux ratio
	AP to BL	BL to AP	
Control	0.81±0.02	1.73±0.11	2.13±0.19
+2 µM elacridar	0.86±0.06	0.88±0.04*	1.02±0.05*
+1 µM Ko143	0.83±0.01	1.07±0.04*	1.26±0.01*
+100 µM verapamil	0.84±0.05	1.29±0.13	1.55±0.16
+10 µM MK571	0.73±0.02*	0.81±0.05*	1.11±0.06*

Transcellular transport of uric acid in Caco-2 monolayer was measured in the absence or presence of elacridar (2 µM), Ko143 (1 µM), verapamil (100 µM) and MK571 (10 µM) at 37°C and pH 7.4. The ratio was obtained by dividing BL-to-AP permeability by AP-to-BL permeability. The values indicate mean ± S.E.M. (n = 3). An asterisk (*) and shows a significant difference from the control by Student's t-test (*p*<0.05).

## Discussion

In this study, we investigated the contribution and molecular mechanism of extra-renal elimination of uric acid. Since elevated SUA has been reported to be associated with several diseases [3], [4], an understanding of the mechanism of uric acid disposition is important in order to control SUA level. In humans, it is reported that two-thirds of uric acid is excreted from kidney, while the rest is recovered in feces [24]. In order to examine whether uric acid is eliminated into intestinal lumen in rats, as it is in humans, we firstly measured the extent of ^14^C recovery after intravenous injection of [^14^C]uric acid in oxonate-treated rats as a model animal (Table 1). Uric acid was excreted into bile and intestinal lumen in addition to urine, and most of the uric acid is secreted directly into the intestinal lumen from blood, but not via the bile ducts, as an extra-renal elimination pathway.

In order to confirm that intestinal luminal secretion is the major extra-renal elimination pathway, we further evaluated intestinal secretion of endogenous uric acid by means of the *in situ* intestinal closed loop method in oxonate-treated rats (Table 2 and Fig. 1A). The estimated total intestinal clearance was 0.15 mL/min (Table 2), which was less than the urinary excretion clearance, 0.50 mL/min, but much larger than the biliary excretion clearance, 0.02 mL/min. The relative contribution of extra-renal excretion versus urinary excretion was comparable to that in the case of ^14^C recovery *in vivo* (Table 1). This demonstrates a significant contribution of intestinal secretion to extra-renal elimination. Furthermore, endogenous uric acid was secreted from blood directly into the intestinal lumen at all intestinal segments, and the secretion at the ileum was about three- and two-times greater than in jejunum and colon, respectively (Fig. 1A).

Then, we examined the molecular mechanism(s) of extra-renal excretion of uric acid into intestinal lumen. Recently, Matsuo *et al.* reported that genetic polymorphisms resulting in decreased BCRP activity in humans are associated with an elevated SUA level, resulting in an increased incidence of gout [36]. BCRP is expressed in the apical membrane of proximal tubular cells in humans [29], suggesting that it plays a role in urinary excretion of uric acid. Since BCRP expression is much higher in the intestinal epithelial cells and hepatocytes than in proximal tubular cells [37], we compared the roles of BCRP and other transporters, MRP2 and MDR1, in extra-renal excretion of uric acid. These transporters are well-characterized efflux transporters for xenobiotics and drugs in both intestine and liver. Uric acid was transported by hBCRP/rBcrp and rMrp2, but not by hMRP2 or hMDR1 (Fig. 2). Since rMdr1a-expressing membrane vesicles were not available, we examined bidirectional transport of uric acid in LLC-PK1 cells expressing rMdr1a; however, no significant difference between secretory and absorptive transport of uric acid was observed (data not shown). Accordingly, it appears that uric acid is a substrate of rBcrp, as well as hBCRP [18], [36], and rBcrp and hBCRP mediate uric acid secretion directly into the intestinal lumen and via the bile duct. In further study, rBcrp-mediated transport of uric acid was strongly inhibited by elacridar, Ko143 and estrone-3-sulfate, and moderately by benzbromarone, MK571, rifampicin and cyclosporin A, respectively, but was not affected by ketoprofen and salicylic acid (Fig. 2C). These results are in agreement with the previous observations in hBCRP-mediated transport [35], [38], [39]. Furthermore, K_m_ value of uric acid by rBcrp, 12.5 mM, was relatively close to that by hBCRP, 8.24 mM [36]. Accordingly, uric acid transport by BCRP shows similar characteristics between human and rat. Because rMrp2 is expressed in the bile canalicular membranes and intestinal apical membrane [40], [41], rMrp2, but not hMRP2, may also be involved intestinal secretion of uric acid.

Since rBcrp was suggested to mediate uric acid secretion into the intestinal lumen, the effect of the rBcrp inhibitor elacridar was examined. Secretion of endogenous uric acid into the intestinal lumen was significantly reduced in the presence of 10 µM elacridar at all intestinal segments (Fig. 1A). Furthermore, the inhibitory potential of elacridar on the uric acid uptake in rBcrp-expressing vesicles was comparable to that on the intestinal clearance of uric acid at rat ileum. Additionally, the mRNA expression level of rBcrp in each segment was comparable with the regional differences of intestinal secretion in the segments (Fig. 2). These findings strongly indicated that Bcrp is at least involved in the direct secretion of uric acid from intestines.

To obtain further evidence that Bcrp contributes to intestinal secretion of uric acid, we employed intestinal closed loop assay of oxonate-treated *Bcrp* ^−/−^ mice. Plasma uric acid was significantly higher in *Bcrp1^−/−^* mice and ileal secretory clearance of uric acid was significantly lower in *Bcrp1^−/−^* mice, as compared with wild-type mice. These results confirmed that Bcrp is an important player in uric acid disposition. However, in rodents, Bcrp1 expression in kidney relative to intestine and liver is reported to be comparable [30]. Therefore, urinary excretion of uric acid was also reduced as well as intestinal secretion in *Bcrp1^−/−^* mice. Further, a compensatory mechanism may operate in *Bcrp1^−/−^* mice, because the SUA level should be kept constant physiologically in rodents as well as in humans. Indeed, no significant change of SUA level was observed in mice lacking the uric acid reabsorptive transporter Urat1 [42]. Therefore, it may not be reasonable to evaluate the quantitative contribution of Bcrp based only on the results in transporter-gene deficient mice.

Finally, we evaluated the intestinal secretion of uric acid in humans using Caco-2 cells as a model of human intestinal epithelial cells. Secretory transport of uric acid across Caco-2 cell monolayer was greater than its absorptive transport, and such polarized transport was abolished in the presence of elacridar, Ko143 and MK571 (Table 3). These results imply that uric acid is secreted into the intestine through the intestinal epithelial cells in human as well as rat, and this process could be mediated by an efflux transporter BCRP in human. Since MK571 and verapamil were reported to slightly inhibit BCRP [35], uric acid transport might be reduced by them. Unexpectedly, bidirectional transport of uric acid was dramatically reduced in the presence of MK571, which inhibits effectively MRP members. Although further clarification of the mechanism should be warranted, this observation may involve other transporters in uric acid transport expressed in Caco-2 cells rather than BCRP, such as MRP4, which is a putative uric acid transporter.

Our results indicate that extra-renal excretion of uric acid amounts to at least one-fourth of urinary excretion in rats, which is comparable with the contribution in humans, and this process cannot be ignored in considering uric acid disposition. Furthermore, the direct intestinal secretion of uric acid was much greater than biliary excretion, suggesting that extra-renal elimination of uric acid mainly involves direct secretion into the gut lumen through the intestinal epithelial cells from blood, but not through the bile ducts. In humans, it has been reported that the uric acid concentration in bile is 1–2 mg/dL, and biliary clearance is 0.1–0.3 mL/min [43]. Therefore, biliary excretion of uric acid should be a minor contributor to the extra-renal elimination pathway in humans as well as in rats, because urinary clearance is about 5.8–6.3 mL/min in humans [44], [45]. Furthermore, it was directly demonstrated for the first time that hBCRP/rBcrp is likely to contribute to intestinal and biliary excretion of uric acid.

Accordingly, it may be possible to decrease the SUA level in hyperuricemia/gout patients by enhancing intestinal elimination of uric acid through activation of BCRP function. It has been reported that sevelamer, a non-absorbable hydrogel, adsorbs uric acid nonselectively in the gastrointestinal tract, and decreases SUA in humans [46], [47]. This is consistent with the idea that the SUA level can be controlled by enhancing secretion of uric acid into intestinal lumen.

## Materials and Methods

### Chemicals and animals

[^14^C]Uric acid (1.96 TBq/mol) was purchased from Moravek Biochemicals, Inc. (Brea, CA, U.S.A.). Elacridar and potassium oxonate were purchased from Toronto Research Chemicals (North York, Ontario, Canada) and Kanto Chemicals (Tokyo, Japan), respectively. All other reagents were purchased from Sigma-Aldrich (St. Louis, MO, U.S.A.), Wako Pure Chemical Industries, Ltd. (Osaka, Japan), Nacalai Tesque (Kyoto, Japan), and Applied Biosystems (Foster City, CA).

All the animal studies were approved by the Committee of Kanazawa University for the Care and Use of Laboratory Animals and were carried out in accordance with its guideline for the Care and Use of Laboratory Animals (AP-111937, AP-111938 and Mm687). Six-week-old Wistar rats were purchased from Sankyo Labo Service Corporation, Inc. (Tokyo, Japan). For the *Bcrp1^−/−^* mouse study, mice were obtained from Taconic Inc. (Hudson, NY).

### *In vivo* animal study

Male Wistar rats (280±30 g body weight) used in the present study were treated with potassium oxonate, a uric acid oxidase (uricase) inhibitor. Oxonate-treated rats were established as described previously [34], [48]. Briefly, rats were anesthetized with 50% urethane (2 mL/kg) and infused with 0.9% NaCl, 0.5% oxonate and 4% mannitol solution from the femoral vein at the flow rate of 3 mL/hr. As a bolus, 0.9% NaCl, 1.5% oxonate and 4% mannitol solution was administered from the jugular vein at 6 mL/kg. After equilibration for 80 min, blood, urine and bile samples were collected at 20-min intervals. When [^14^C]uric acid (2 µCi/rat) was used, it was intravenously injected from the jugular vein after oxonate treatment for 80 min. In order to suppress the uricolysis of [^14^C]uric acid by intestinal flora [24], animals received orally non-absorbed antibiotics, polymyxin B, kanamycin, and vancomycin at daily doses of 50, 50 and 20 mg/kg, respectively, for 7 days before this animal study. Urine and bile were collected at 20-min intervals, and blood samples were drawn at 1, 5, 10, 20, 40, 60, 80, 100 and 120 min. Urine and bile were collected from bladder and bile ducts, respectively, and blood from the jugular vein. In mice, urine was collected for 24 hours by using metabolic cages (Natsume, Tokyo, Japan).

### Intestinal luminal secretion study by *in situ* intestinal closed loop method

We evaluated intestinal secretion of endogenous uric acid according to the method described previously, with some modifications [49]. Rats were anesthetized with 50% urethane and treated with oxonate by the same method as in the *in vivo* animal study described above. Intestinal closed loops were made at different segments: jejunum (10 cm), ileum (10 cm), and colon (3–5 cm). Phosphate-buffered solution (PB, 0.075 M NaH_2_PO_4_ - 0.075 M Na_2_HPO_4_, pH 6.5) or inhibitor-containing PB was injected into the intestinal loop. After 60 min, the remaining luminal content in each loop was washed out and collected for quantitation. Blood was obtained from the jugular vein at 1, 20, 40 and 60 min. Plasma was obtained by centrifugation and used for quantitation.

Mice were treated with oxonate (intraperitoneal injection of 200 mg/kg oxonate in 1% arabic gum solution) at 1 hr before injection of PB solution into the intestinal loop. Blood was withdrawn from the jugular vein at 1, 30 and 60 min. At 60 min, the intestinal luminal content was collected for measurement of intestinally secreted uric acid.

The following equation was used to calculate intestinal secretory clearance (µL/min/cm loop) and estimated total intestinal secretory clearance (CL_intestine, estimated_) (mL/min):
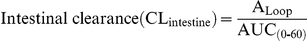


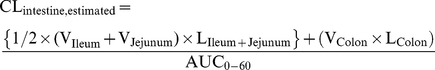


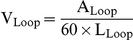
where A is the amount of uric acid secreted into the intestinal lumen of each loop for 60 min, L is the length of the intestine, and AUC is area under the plasma concentration-time curve.

### Transport study in membrane vesicles

hBCRP/rBcrp, hMRP2/rMrp2, or hMDR1-expressing membrane vesicles prepared from Sf9 cells were purchased from GenoMembrane Inc. (Yokohama, Japan). Uptake experiments were conducted as described previously [50]. Briefly, after a pre-incubation for 5 min at 37°C, membrane vesicles (50 µg protein/50 µL final reaction volume) were incubated for 5 min at 37°C in the presence of 4 mM ATP or AMP in assay buffer (1 M KCl, 1 M MgCl_2_, and 100 mM MOPS-Tris, pH 7.0) containing [^14^C]uric acid (1 µCi/mL). The uptake reaction was terminated by adding 1 mL ice-cold washing buffer (1 M KCl, and 100 mM MOPS-Tris, pH 7.0) to the membrane solution, and the mixture was rapidly filtered through a nitrocellulose filter (0.45 µm pore size, Millipore, Bedford, MA). The filters were washed twice with 5 mL ice-cold washing buffer. The filters were dried and dissolved in Clearsol-I for quantitation of radioactivity.

### Bidirectional trans-cellular transport study in Caco-2 cells

Caco-2 cells were obtained from the American Type Culture Collection (Rockville, MD). Caco-2 cells were cultured in Dulbecco's modified Eagle's medium (D-MEM) (Invitrogen, Carlsbad, CA) supplemented with 10% (v/v) fetal bovine serum (Invitrogen), 100 units/mL benzylpenicillin, 100 µg/mL streptomycin, 1% (v/v) MEM non-essential amino acid solution (Invitrogen) at 37°C under an atmosphere of 5% CO_2_ in air. For bidirectional transport studies, Caco-2 cells were cultured on Transwell filter membrane inserts (BD Falcon, surface area 0.9 cm^2^ and pore size 3 µm) at a density of 6.4×10^4^ cells/cm^2^ for 21 days before use for each experiment. Transport measurement was initiated by adding transport medium (5.36 mM KCl, 136.89 mM NaCl, 0.34 mM Na_2_HPO_4_⋅7H_2_O, 0.44 mM KH_2_PO_4_, 4.17 mM NaHCO_3_, 1.26 mM CaCl_2_, 0.49 mM MgCl_2_⋅6H_2_O, 0.41 mM MgSO_4_⋅7H_2_O, 19.45 mM glucose, 10 mM HEPES/Tris, pH 7.4) containing [^14^C]uric acid to the donor side and transport medium without [^14^C]uric acid to the receiver side. Inhibitor was added to only apical side. Transport of [^14^C]uric acid was measured in two directions, namely apical (AP)-to-basal (BL) and BL-to-AP directions. An aliquot of transport buffer was obtained from the donor side at 5 min for measurement of initial concentration, and from the receiver side at 30, 60, 90 and 120 min. Transport studies were performed at 37°C and pH 7.4.

The apparent permeability (P_app_, cm/sec) of [^14^C]uric acid across the cell monolayer was calculated using the following equation:
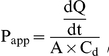
where Q is the amount of [^14^C]uric acid transported over time t. C_d_ is the initial concentration in the donor side and A is the surface area of membrane.

### Real-time reverse transcription polymerase chain reaction

Total RNA was prepared from intestinal mucosa by using standard methods. Single-strand cDNAs were synthesized with a High Capacity cDNA Reverse Transcription Kit (Invitrogen). Relative quantification of rat Bcrp and rat glyceryl aldehyde 3-phosphodehydrogenase (Gapdh) mRNA expression was performed with a Real-Time PCR system (Mx3000p, Stratagene, Cedar Creek, TX) using Brilliant SYBR Green QPCR Master Mix and Reference Dye (Stratagene) with 35 cycles of denaturation at 95°C for 15 sec, annealing at 58°C for 20 sec, and extension at 72°C for 20 sec. The expression level was normalized to that of rat Gapdh. The sequences of gene-specific primer pairs were follows: forward and reverse primers for rBcrp are 5′-TTTGATAAACGGGGCACCTC-3′ and 5′-AGCTTTTGGAAGGCGAAGAG-3′, respectively, and for Gapdh are 5′-GGTGGACCTCATGGCCTACA-3′ and 5′-ATTGTGAGGGAGATCCTCAGTGT-3′, respectively.

### Analytical methods

Concentrations of uric acid in plasma, urine, bile, and intestinal content were analyzed by means of HPLC. Briefly, 80 µL 0.41% HClO_4_ was added to 40 µL of sample and mixed well. The sample was kept 4°C for 30 min. An aliquot was centrifuged at 15,000 rpm for 5 min at 4°C to separate denatured proteins. Eighty µL of the supernatant was mixed with an equal amount of 200 mM Na_2_HPO_4_ solution, and then a portion (50 µL) was applied to the HPLC system (Alliance 2690/UV/VIS Detector 486, Waters,Milford, MA). The HPLC analysis was performed using Mightysil RP-18 GP 5 µm (250 mm×4.6 mm, Kanto Chemical Co. Tokyo, Japan) as an analytical column at 30°C. The mobile phase was composed of 40 mM Na_2_HPO_4_ (pH 2.55)/methanol (99/1), delivered at a flow rate of 1 mL/min. The retention time was about 7.0–7.5 min. Uric acid was detected at 284 nm. The calibration curve was linear over the range of 0.25–50 mg/L.

All data are presented as the mean ± S.E. Statistical significance was evaluated with Student's t-test with *p*<0.05 as the criterion for a statistically significant difference.
